# Research on adsorption characteristics of H_2_S, CH_4_, N_2_ in coal based on Monte Carlo method

**DOI:** 10.1038/s41598-020-78927-6

**Published:** 2020-12-14

**Authors:** Jiren Wang, Cong Ding, Dameng Gao, Hongpeng Liu

**Affiliations:** 1 College of Safety Science & Engineering, Liaoning Technical University, Fuxin, 123000 Liaoning China; 2 Key Laboratory of Mine Thermodynamic Disaster & Control of Ministry of Education, Huludao, 125105 Liaoning China; 3 Datong Coal Mine Group Co., Ltd., Ventilated Place Datong, Shanxi, 037003 China

**Keywords:** Solid Earth sciences, Energy science and technology

## Abstract

In order to study the adsorption characteristics of H_2_S, CH_4_ and N_2_ by coal under different conditions, the new macromolecular structure model of Dongqu No. 2 was constructed, and the grand canonical Monte Carlo (GCMC) method was used to simulate the adsorption process of three types of gases in coal. The dependence of adsorption capacity of coal on its temperature, pressure and moisture content was analyzed. The results show that with the increase of pressure and temperature, adsorption isotherms of all the three gases follow Langmuir model. For pressure greater than 2 MPa, the influence of temperature on adsorption capacity was greater than that of pressure. With rise in temperature, the decrease in rate of H_2_S adsorption was least and drops in the heat of adsorption of H_2_S most. This indicates that the adsorption of H_2_S on coal is more stable than those of CH_4_ and N_2_. As the water content of coal increased, its adsorption capacity for the present three gases decreased linearly, and the capacity for H_2_S (1.77 mmol/g) changed the most. The reduction of free volume linearly and preferential occupation of adsorption sites by water molecules are the main reasons for the highest change in the adsorbed amount of H_2_S gas.

## Introduction

In the mining of high-sulfur coal, the escape of a toxic and harmful H_2_S gas threatens the safety of health and life of coal miners^1,2^. It is reported that inhaling by human being of H_2_S gas at a concentration level 1 g/m^3^ can cause death within seconds^3–5^. More than 10 deaths due to poisoning by H_2_S gas have been reported in our country in the present century. With gradual exhaustion of earth's fossil resources of energy, the depth of coal mining is expected to increase, and consequently H_2_S is going to become a major concern of safety in coal production^6–8^. Therefore, understanding of adsorption characteristics of coal for H_2_S gas is of significance currently.

The origin of H_2_S gas in coal is a subject of research globally^9–11^. It is believed currently that the gas existing with coal has come from three processes: biochemical including biodegradation and microbial sulfate reduction (BSR); thermochemical, including thermochemical decomposition (TDS) and thermochemical sulfate reduction; and Magma. The toxic nature of H_2_S, limits experimental studies on it. The main factors influencing adsorption of H_2_S on coal are: pores, microscopic components, moisture, pressure, degree of coalification etc^12^. Based on identical experiments carried out on isothermal adsorption of H_2_S, CH_4_ and N_2_ on coal. He et al.^13^ reported that highest adsorption capacity of coal is for H_2_S (among the present three gases) and the lowest for N_2_. Liang et al.^14^ simulated the adsorption of H_2_S, CO_2_, CH_4_, and N_2_ gas molecules on the coal surface by quantum chemical methods, and reported that the adsorption capacity for H_2_S is lowest, and the presence of H_2_S in the coal seam enhances its ability to adsorb CH_4_. Xue et al.^15^ studied the interaction between functional groups present on coal and H_2_S molecules using molecular simulation, and found that between hydroxyl group and H_2_S the interaction is strongest. Xue et al.^16^ studied adsorption of H_2_S gas on different types of coal samples and used adsorption models suited to them. The results have shown that adsorption capacity of coal for H_2_S increases with the degree of coalification, and Langmuir model is the best fit. Many research groups globally, have studied in detail adsorption of CH_4_ and N_2_ on coal^17–22^, and therefore CH_4_ and N_2_ have been selected in the present work as controls to analyze the data of adsorption of H_2_S on coal. Monte Carlo method applied to understand adsorption of various gases on coal in the recent past has been used here for calculations^23–26^.

From the above research, we can find that the previous research is mainly based on experiments. The adsorption experiments only measure and analyze the adsorption phenomenon at a macro level, and cannot describe the microscopic process of adsorption. The computational methods simulating adsorption on coal are not disturbed by the external environment. The experimental research has laid undoubtedly some theoretical basis to discuss adsorption of H_2_S on coal but the adsorption characteristics of H_2_S are scantly analyzed from a microscopic point of view. The effect of water associated with coal on adsorption of H_2_S also has not been studied. The level of water content in coal is an important factor that affects the amount of H_2_S adsorbed. In the present paper, the adsorption characteristics of coal for H_2_S gas are studied from the perspective of macromolecular organic structure of coal, and the adsorption characteristics of CH_4_ and N_2_ are used as a control to analyze the difference in the adsorption capacity of coal for the present three single-component gases. This paper uses molecular simulation methods to study adsorption of H_2_S, CH_4_ and N_2_ (as a single-component gas) on coal, Dongqu No. 2, use of which eliminates the influence of different geological conditions on the inorganic contents of coal. The effect of different pressures (up to10MPa), temperatures (298, 318, 338, 358 and 378 K) and water contents (0, 1.18, 2.31, 3.42, and 4.51%) on the adsorption isotherm curve, isosteric heat of adsorption and energy of adsorption are reported. Thus, a theoretical basis is provided for control of H_2_S in coal seams.

## Dongqu No. 2 coal model and simulation method

**Figure 1:**
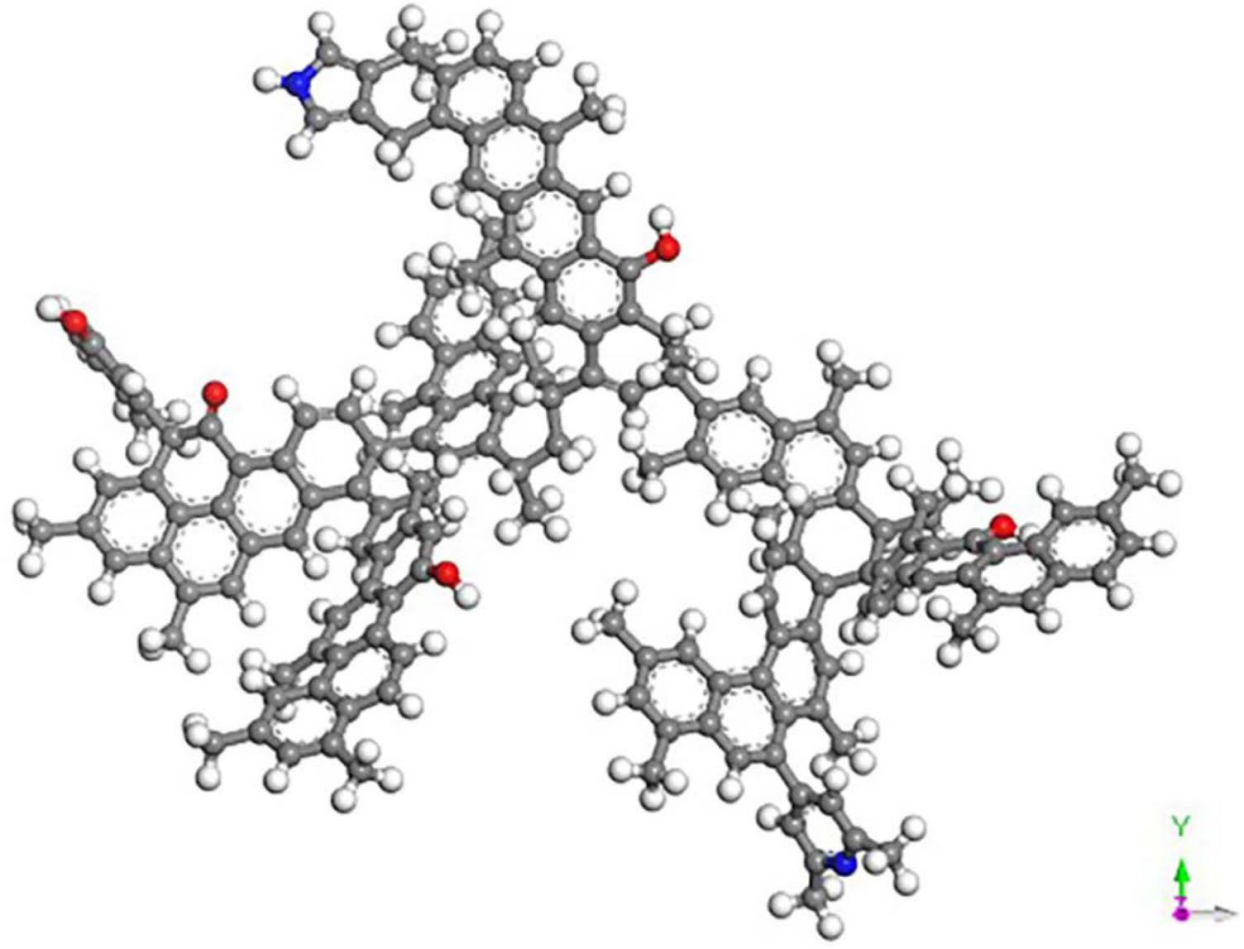
Molecular model of Dongqu No.2.

**Figure 2:**
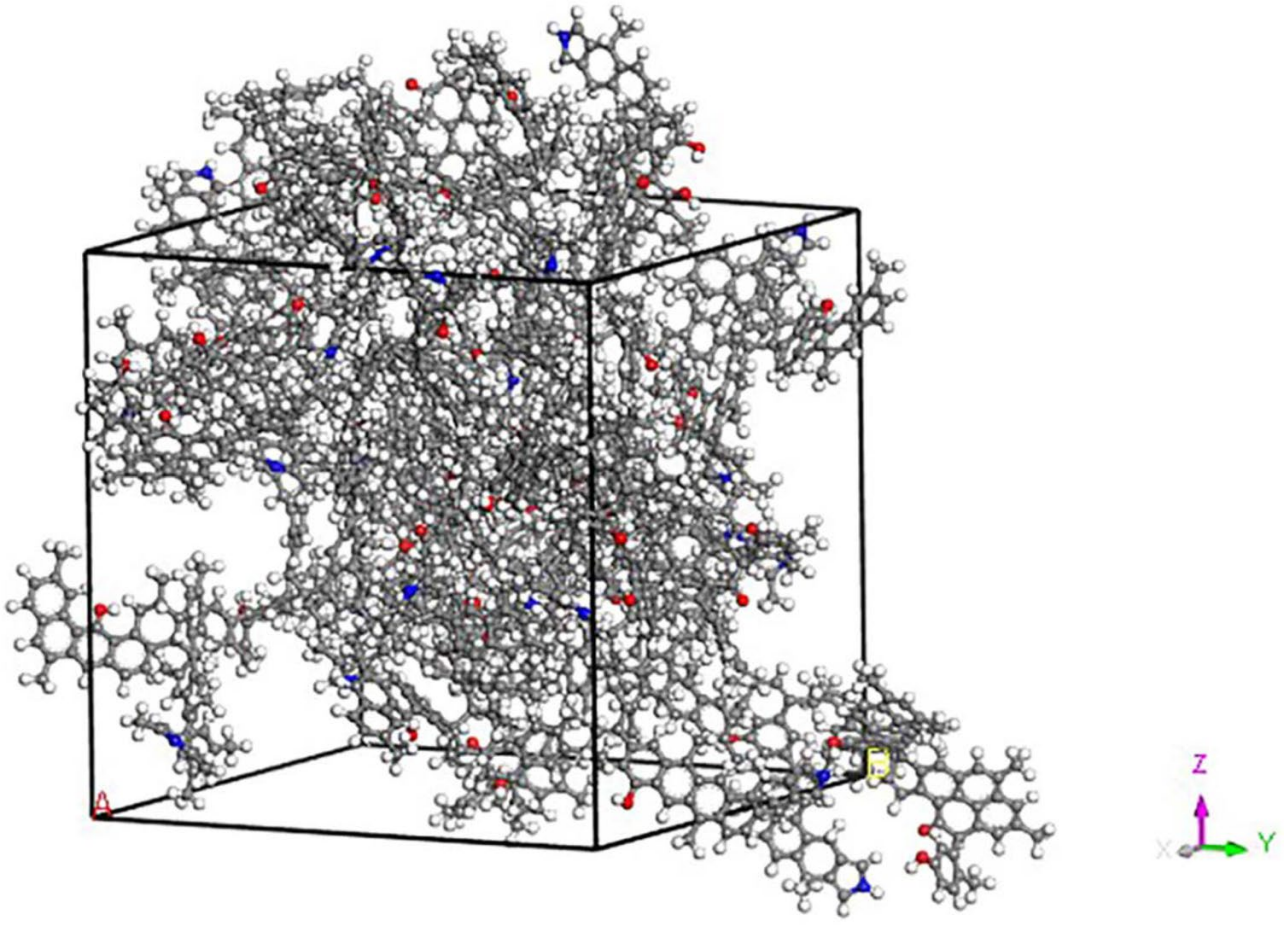
Model of coal structure.

The molecular structure model of Dongqu No. 2 (Fig. 1) proposed by Li Yaogao^27^ for coal was adopted in the present paper. The aromatic skeleton of this model is mainly anthracene ring, and it contains phenolic hydroxyl, carboxyl, pyridine and pyrrole functionalities. Its molecular formula is C_174_H_148_O_5_N_2_.The molecular model constructed for coal has used the “Forcite” module to optimize its geometry and energy. The lowest energy of the molecular model is shown in Fig. 1. It uses the module Amorphous Cell to put thirteen molecules of coal in the box, and COMPASS force field is selected for geometric optimization of the constructed three-dimensional structure, annealing, and molecular dynamics^28^. As the constructed coal model has a periodic structure, the electrostatic interactions are all Ewald and van der Waals type and are based on atom. The molecular dynamics parameter settings are shown in Table 1. The final model is shown in Fig. 2.

**Table 1:** Parameters of molecular dynamics.

Force field	COMPASS^28^	Charges	Use current
Electrostatic	Ewald^29^	Production	10^6^
Van der Waals	Atom based^30^	Step size/fs	1
Canonical ensemble	NPT^31^	Steps total time/ps	1000
Temperature	Nose^32^	Quality	Fine

Based on the coal structure model constructed (Fig. 2), the Sorption modules were used to calculate at fixed pressure (0.01, 2, 6, and 8 MPa) adsorption isotherms (298, 318, and 338 K). The Sorption Locator module was used to build coal structure models with different moisture content. Thereafter to simulate the gas adsorption Sorption module was used. The average loading (Eq. 1) is the amount adsorbed. Its unit is N/uc, and conversion formula for the unit is:

1 $${\text{uptake/(mmol/g}}) = \frac{{\text{average loading}}}{{\text{M}}} \times {10}^{{3}}$$

M is the relative molecular mass of the adsorbent (g/mol).

## Results and analysis

### Adsorption isotherm

**Figure 3:**
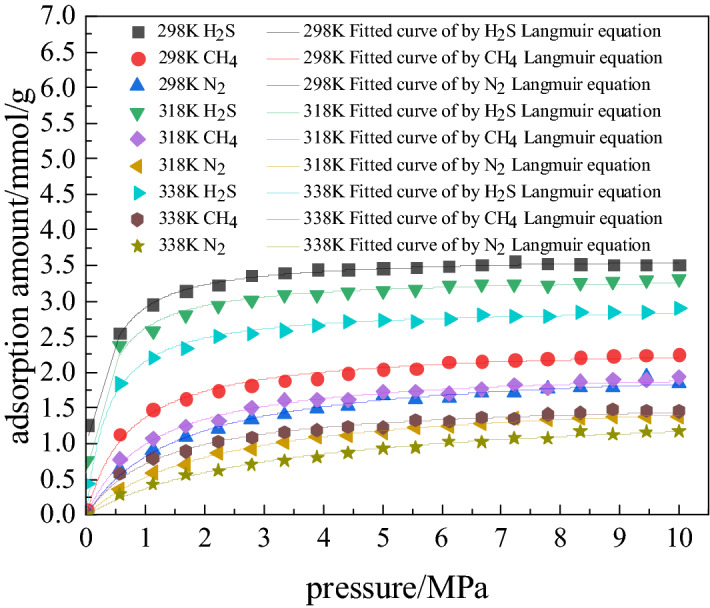
Adsorption isotherms of H_2_S, CH_4_ and N_2_ at different temperatures.

The adsorption isotherms of coal molecules at temperatures 298, 318, and 338 K for H_2_S, CH_4_ and N_2_ are shown in Fig. 3. It reveals that pressure has a very large effect on the adsorption capacity of coal for the three gases. With the increase in pressure, the adsorption capacity of coal for the three gases first increases rapidly and then slowly. At different temperatures, the adsorption curves of the three gases show similar trend. The temperature affects only the quantity of gas adsorbed, and does not change the trend of its adsorption. Langmuir model (Eq. 2) is followed by adsorption of each gas (Fig. 3)

2 $$Q = \frac{abp}{{1 + bp}}$$

Here Q: is adsorption capacity for gas (mmol/g); a, maximum adsorption capacity of coal sample (mmol/g); b, adsorption constant, (MPa^−1^); p, pressure of gas adsorbed, (MPa).

The fitting constants and correlation coefficients of three gases at different temperatures are shown in Table 2.

**Table 2:** Langmuir parameters of adsorbed gases at different temperatures.

Temperature	298 K			318 K			338 K		
	a	b	R^2^	a	b	R^2^	a	b	R^2^
H_2_S	3.4521	3.9335	0.9911	3.3149	3.3955	0.9571	2.9157	2.4730	0.9741
CH_4_	2.3681	1.3745	0.9910	2.0831	0.9086	0.9925	1.6199	0.7844	0.9903
N_2_	2.1303	0.6227	0.9915	1.7203	0.4394	0.9973	1.5386	0.3120	0.9925

Figure 3 shows that at some pressure, the adsorption capacity of coal for H_2_S, CH_4_ and N_2_ decreases with increase in temperature. It can be seen from the above figure, the influence of pressure in the range 0–2 MPa on the adsorption capacity of coal for H_2_S, CH_4_ and N_2_ is greater than influence of temperature on adsorption.

At a pressure of 10 MPa, average reduction rate of adsorption of H_2_S, CH_4_, and N_2_ on coal with rise in temperature is 13, 14, and 20%, respectively. The adsorption of N_2_ is most affected by temperature, followed by CH_4_ and H_2_S. This may be correlated to their critical temperatures which are 212.75, 111.65, and 77 K for H_2_S, CH_4_ and N_2_ respectively. The gas with high critical temperature liquefies easily and is adsorbed on coal matrix^33^. At a particular temperature, repulsion between molecules of H_2_S is least and capacity of coal for its adsorption is highest in comparison to those of two other gases investigated here.

**Figure 4:**
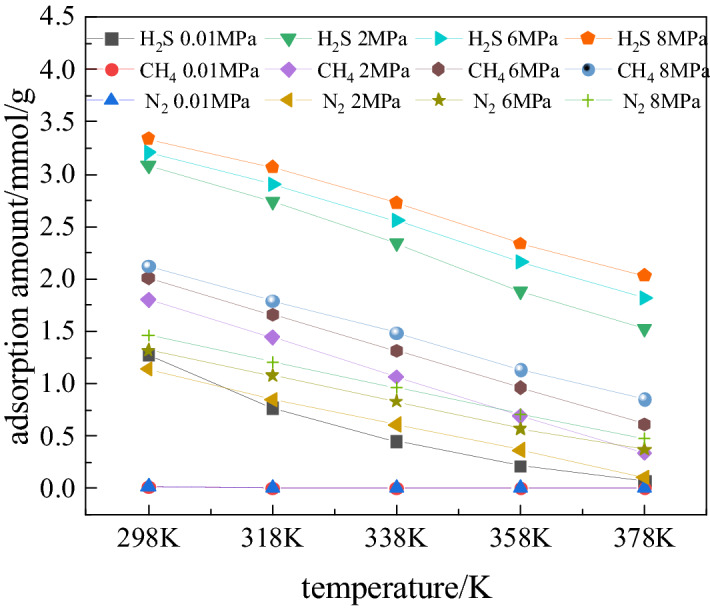
Adsorption capacity of H_2_S, CH_4_ and N_2_: variation with temperature at different pressures.

In order to analyze the effect of temperature on the adsorption capacity of coal for the three gases, considered Fig. 3. In the initial stage of adsorption, the pressure (up to 2 MPa), shows a large effect on the adsorption capacity of the gases, but the effect of temperature on adsorption capacity of coal for the three of gases is not regular. Therefore, the relationship between adsorption capacity and temperature was analyzed at fixed pressures 2, 6 and 8 MPa (Fig. 4). As shown in Fig. 4, in the temperature range 298–378 K and pressure at 2 MPa, the adsorption capacity (mmol/g) of coal for H_2_S, CH_4_ and N_2_ drops by 1.55, 1.43 and 1.04 respectively. At 8 MPa, and in the same temperature range, the capacity (mmol/g) for H_2_S, CH_4_ and N_2_ drops by 1.30, 1.26 and 0.98 respectively. In the pressure range 2-8 M Pa, and at temperature 378 K, adsorption capacity of coal changes by 0.51 mmol/g for H_2_S; 0.48 mmol/g for CH_4_, and 0.38 mmol/g for N_2_. Thus, the temperature also has a high influence on the adsorption capacity of coal for the three gases at pressure beyond 2 MPa.

### Isometric heat of adsorption

**Figure 5:**
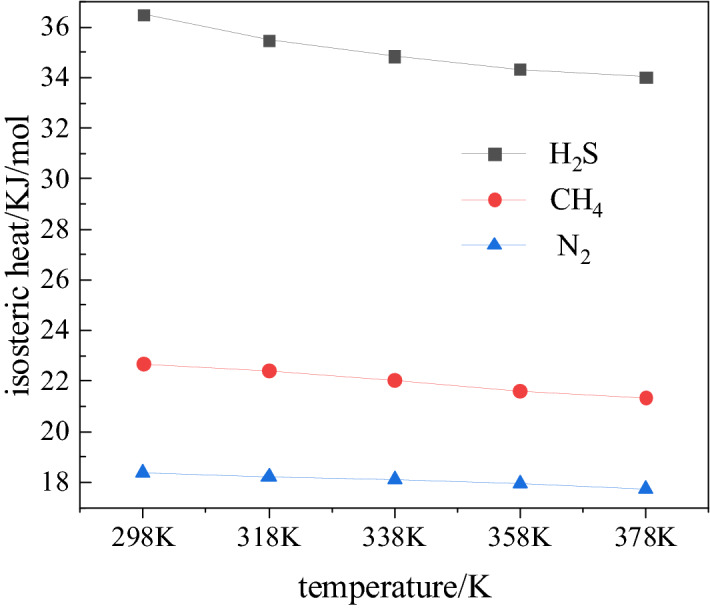
Adsorption heat of H_2_S, CH_4_ and N_2_: Variation with temperature.

The heat of adsorption indicates the strength of adsorption. In the adsorption process, gas molecules move towards the surface of coal, and the speed of their molecular motion is reduced greatly, and thus heat is released^34,35^. The relationship between heat of adsorption of H_2_S, CH_4_ and N_2_ with temperature is shown in Fig. 5. The heat decreases with increase in temperature. The average gradients of decrease for H_2_S, CH_4_ and N_2_ are 0.44/20 K, 0.27/20 K, 0.13/20 K, respectively. At 298 K, the heat of adsorption on coal for the three gases H_2_S, CH_4_ and N_2_ is highest, 36.49, 22.67, and 18.38 kJ/mol respectively. As all the heats are less than 42 kJ/mol, the adsorption of H_2_S, CH_4_ and N_2_ on coal is of physical type, which is consistent with the earlier results^14^. From the average gradient of the drop of adsorption heat for the three gases, it can be inferred that the adsorption heat of H_2_S decreases the most as the temperature rises. The amount of H_2_S adsorbed is least affected by temperature, thus its adsorption would release more heat than CH_4_ and N_2_, making adsorption more stable.

### Adsorption energy

**Figure 6:**
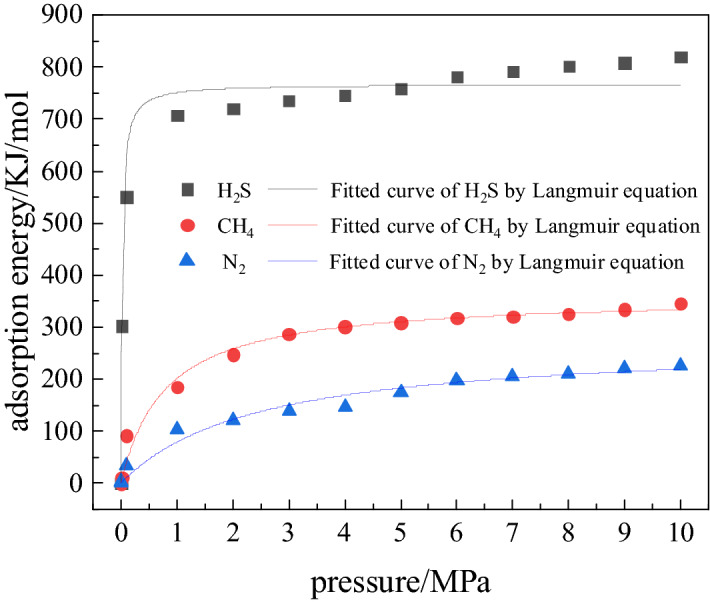
Adsorption energy of H_2_S, CH_4_ and N_2_: variation with pressure.

The adsorption energy referred in this paper includes van der Waals, electrostatic, and intramolecular energies. The intramolecular energy is too small and has been ignored. The van der Waals and electrostatic energy obtained by the simulation are both negative. The numerical value is considered when analyzing the energy strength, thus the absolute value is taken for analysis. Table 3 shows the variation of van der Waals energy and electrostatic energy of adsorption of H_2_S, CH_4_, and N_2_ on coal with pressure at 298 K, and in Fig. 6 also the variation of corresponding adsorption energy with pressure is shown.

**Table 3:** Van der Waals and electrostatic energy of adsorption of each gas at different pressure.

Pressure	Van der Waals			Electrostatic energy		
	H_2_S	CH_4_	N_2_	H_2_S	CH_4_	N_2_
0	0	0	0	0	0	0
0.01	265.55	11.50	4.06	36.91	0	0
0.1	482.95	92.24	35.64	66.86	0	0
1	631.27	185.60	103.74	75.37	0	0
2	639.68	247.13	122.13	80.87	0	0
3	653.34	287.69	139.00	82.58	0	0
4	661.94	301.63	147.95	83.06	0	0
5	673.40	308.99	175.25	84.45	0	0
6	695.14	317.50	198.04	86.95	0	0
7	701.58	320.59	205.52	90.40	0	0
8	703.50	325.37	210.41	99.01	0	0
9	707.98	334.87	220.42	100.21	0	0
10	716.17	345.59	227.08	103.91	0	0

It can be seen from Table 3 that with the increase of pressure, van der Waals energy undergoes significant change. When, the pressure increases from 0 to 10 MPa, the van der Waals energy of H_2_S, CH_4_ and N_2_ adsorption on coal increases by 716.17, 345.59 and 227.08 kJ/mol, respectively. Adsorption of H_2_S on coal releases small electrostatic energy, while such energy released for CH_4_ and N_2_ is 0. This is because H_2_S and coal (due to functional groups) are polar, and force between them is directional. The CH_4_ and N_2_ are non-polar molecules, and the force between them and coal depends on dispersion and inducing.

The fitting constants and correlation coefficients of three gases at different pressures are shown in Table 4. It can be seen from Fig. 6 that as the pressure increases the adsorption energy of H_2_S, CH_4_, and N_2_ first increases rapidly, and then tends to flatten. They all follow the Langmuir adsorption model. When the pressure is 0–2 MPa, the adsorption energy shows a rapid growth and is affected most by the pressure. In the pressure range from 2 to 8 MPa, the energy increases slowly. In slow growth stage, the pressure has little effect on the adsorption energy. This is because the pores on the surface of coal matrix are covered by a large amount of gas, and the gas molecules compete with each other for the remaining vacancies, so that the adsorption capacity increases slowly with the pressure, resulting in slow increases in the adsorption energy. At 8–10 MPa pressure, the adsorption tends to get saturated, i.e. equilibrium stage for adsorption is reached, and the pressure has almost no further effect on the adsorption energy. In this pressure range, the adsorption energy of H_2_S is highest, followed by CH_4_ and N_2_, indicating that the adsorption capacity of coal for H_2_S is highest. In the pressure range of 0–2 MPa, the adsorption energy of H_2_S increases significantly, (by 820.08 kJ/mol), indicating that the pressure has the maximum impact on adsorption of H_2_S at its low value.

**Table 4:** Langmuir parameter of adsorption energy at 298 K.

	a	b	R^2^
H_2_S	767.2742	36.1554	0.9661
CH_4_	359.6122	1.2760	0.9807
N_2_	272.1298	0.4131	0.9721

### Effect of water content of coal

The capacity of coal structure model with moisture content of 0, 0.50, 1.18, 2.31, 3.42 and 4.51% for adsorption of H_2_S, CH_4_ and N_2_ has been calculated at 298 K and 8 MPa pressure respectively, using the following formula (Eq. 3) for water content calculation:

3 $$W = \frac{{M_{{H_{2} O}} }}{{M{}_{{{\text{coal}}}} + M_{{H_{2} O}} }} \times 100\%$$

In the formula, M_H2O_is the molar mass of water (g/mol); M_coal_ is the molar mass of theoretical coal molecule (g/mol).

**Figure 7:**
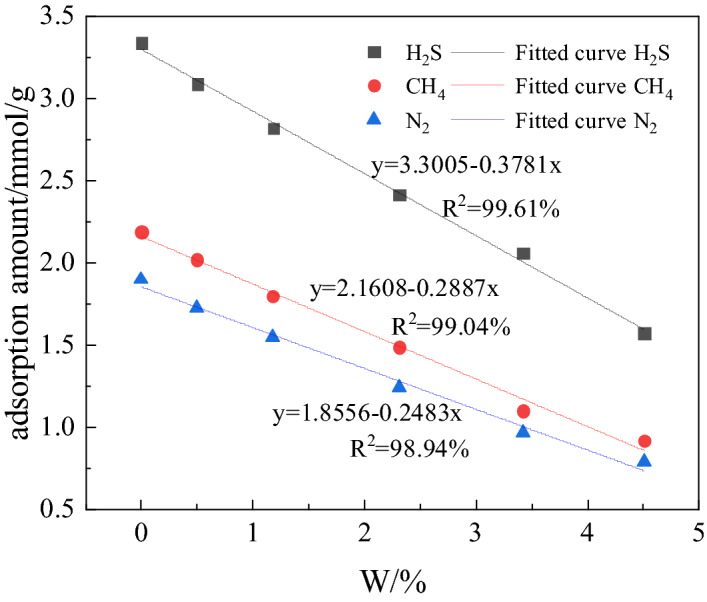
Adsorption of H_2_S, CH_4_ and N_2_: correlation with water present in coal.

**Figure 8:**
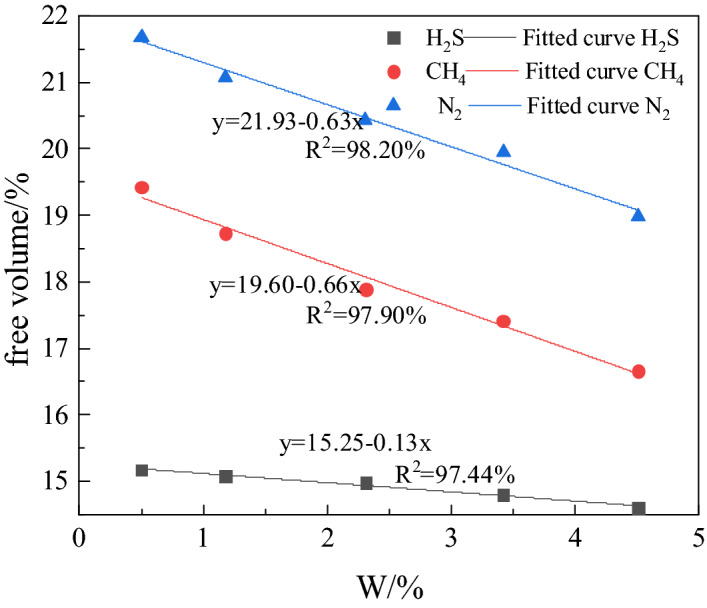
Free volume in coal of varying moisture content after adsorbing gas.

When analyzing the influence of water content on gas adsorption capacity, the number of water molecules added to the simulated coal is 0, 9, 20, 40, 60 and 80. The relationship between the adsorption capacity of coal for the three gases and the moisture content is shown in Fig. 7. It can be seen from the Figure that the adsorption capacity of coal for the three gases decreases with increase in its moisture content. After linear curve fitting, it was found that within the given water content range, the adsorption capacity of coal for H_2_S, CH_4_ and N_2_ decreases linearly with the increase in water content. Linear fitting is 99.61, 99.04 and 98.94%, respectively for H_2_S, CH_4_ and N_2_. It is calculated that, 26.61% of free volume of coal structure model is without water, 25.87% with 9 water molecules, 25.29% with 20 water molecules, 24.14% with 40 water molecules, 22.79% with 60 water molecules and 21.56% with 80 water molecules. The increase in the number of water molecules is accompanied by decrease of free volume in coal, which is the main reason for the decrease in adsorption of gas. In the range of water content 0–4.51%, the adsorption capacity of coal for H_2_S drops from 3.34 mmol/g at 0% water to 1.57 mmol/g at 4.51% of water. The adsorption capacity for CH_4_ drops from 2.19 mmol/g at 0% water to 0.92 mmol/g at 4.51% water, and for N_2_ the drop for the same water content range is from 1.90 to 0.79 mmol/g. The amount of H_2_S adsorbed is reduced the most. Figure 8 reflects the relationship between the free volume of coal and the moisture content after adsorption of gas. It can be seen from Fig. 8 that the free volume of the coal after adsorption of H_2_S is least affected by moisture. Since positions for adsorption of H_2_S and water molecules in coal are the same, water replaces H_2_S from positions of its adsorption. Therefore, the reduction of free volume and the preemption of adsorption sites by water molecules are the main factors causing reduction in adsorption of H_2_S on coal. For CH_4_ and N_2_, as number of water molecules increases, they occupy free volume, which promotes the competitive adsorption of CH_4_ and N_2_. Since the radius of the CH_4_ molecule is larger than that of water, the coal shows a high adsorption capacity for water due to its molecular structure and water occupies the space meant for adsorption of CH_4_, but cannot enter in the space of adsorbed N_2_, which has a smaller radius than that of water molecule. Consequently, ∆Q _(CH4)_ is greater than ∆Q _(N2)_.

**Figure 9:**
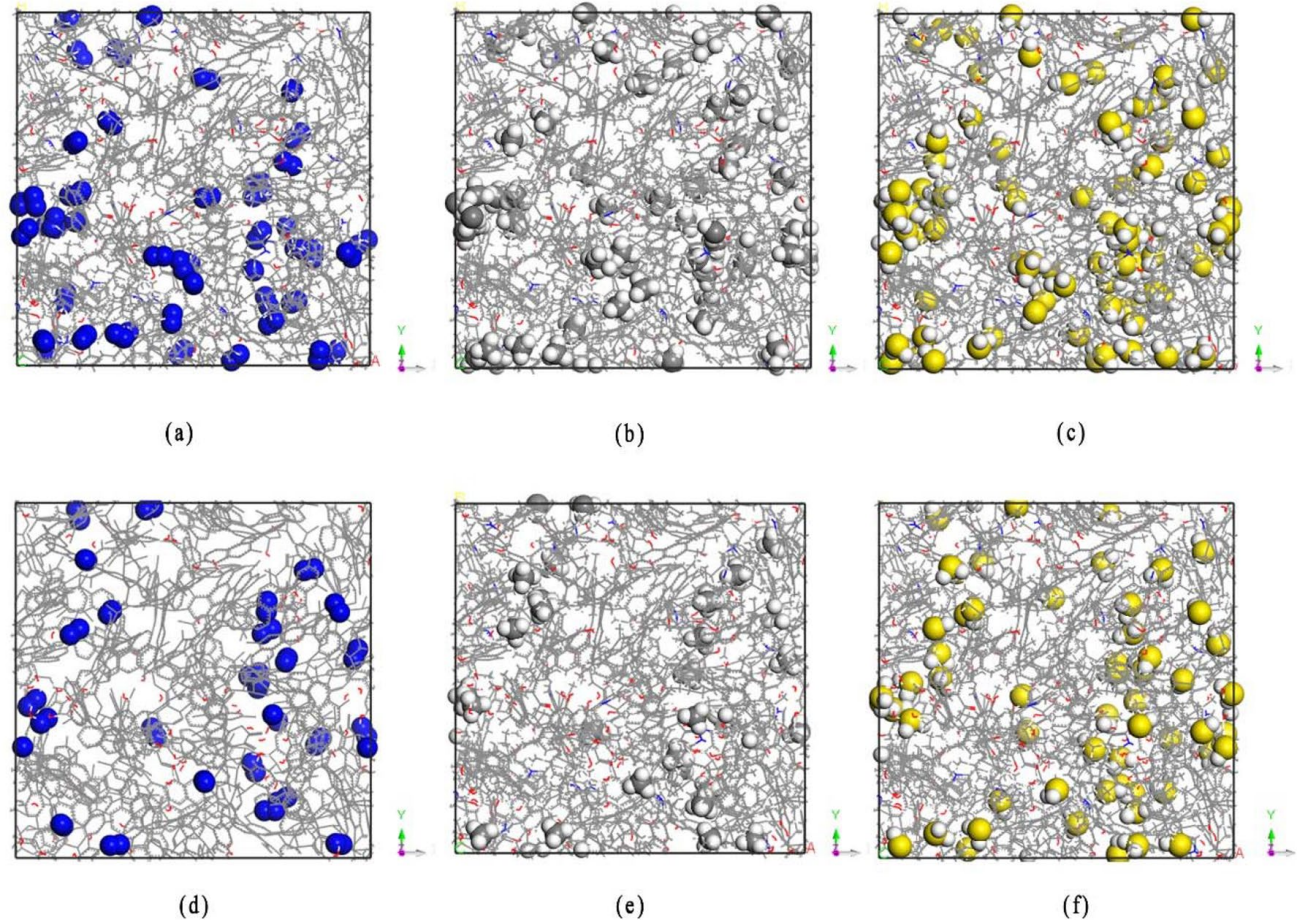
Density distribution of the gases, when content of water in coal is 1.18 or 4.51%. (**a**) N_2_,1.18%, (**b**) CH_4_1.18%, (**c**) H_2_S, 1.18%, (**d**) N_2_, 4.51%, (**e**) CH_4_, 4.51%, (**f**) H_2_S, 4.51%.

In order to intuitively understand the influence of moisture in coal on its adsorption capacity for the present three gases, Fig. 9 shows the density distribution of the three gases when the pressure is 8 MPa and the water content is 1.18 or 4.51%. When the water content is 1.18%, N_2_ is adsorbed sporadically in the coal pores. The H_2_S forms more cluster structures in the pores of coal, compared to CH_4_, indicating high density of adsorption. The number of coal molecules adsorbing H_2_S, CH_4_, and N_2_ are 90, 60, and 46, respectively. When the water content is 4.51%, N_2_ is more dispersed in the coal pores, and H_2_S and CH_4_ are scattered in the pores, indicating that density of adsorption is reduced. At this time, the number of coal molecules adsorbing H_2_S, CH_4_, and N_2_ are 57, 32, and 27, respectively. It can be concluded that as the water content increases, the adsorption density of the three types of gases decreases. The relationship of adsorption capacity H_2_S > CH_4_ > N_2_ is always maintained.

## Conclusions

Based on the results of Monte Carlo method applied to study the adsorption characteristics of H_2_S, CH_4_ and N_2_ in the organic structure of coal under different conditions, the following conclusions can be made:

1. The adsorption isotherms of H_2_S, CH_4_ and N_2_ follow Langmuir model. If pressure is in the range  0-2 MPa, the adsorption capacity of coal for the three gases is more influenced by pressure than temperature. Beyond 2 MPa, the temperature exerts greater influence on the adsorption capacity of coal for these gases than the pressure. When the pressure is the same, the adsorption capacity of coal for the three gases decreases with increase in temperature. The influence of temperature on the adsorption capacity of coal is maximum for N_2_, followed by CH_4_ and H_2_S.
2. The heat of adsorption of the three gases decreases with the increase in temperature, and for H_2_S it drops the most. The amount of H_2_S adsorbed changes the least with the increase in temperature, indicating that the H_2_S adsorbed coal is stable. At 298 K, the heat of adsorption of H_2_S, CH_4_, and N_2_ respectively are 36.49, 22.67 and 18.38 kJ/mol.
3. In the pressure range ~ 0–10 MPa, the adsorption energy of H_2_S is followed by that of CH_4_ and N_2_. The low pressure has the greatest impact on the adsorption energy of H_2_S.
4. At a fixed pressure and temperature, as the number of H_2_O molecules increases, the free volume in the coal decreases proportionately and linearly. The adsorption capacity of coal for the three gases also decreases linearly. The change in the amount of H_2_S adsorbed is 1.77 mmol/g. The linear decrease in the free volume and preferential occupation of adsorption sites by water molecules are the main reasons for the largest change in the amount of H_2_S gas adsorbed.

## References

[1] Fu XH, Liu AH, Wang KX, Shen J (2011). Prevention and origin of exceptional deleterious gas compositions in coal mine. Procedia Eng..

[2] Deng P, Cheng B, Yang S, Peng MH (2019). Research progress on the determination method of hydrogen sulfide content in underground coal seams. Coal Mine Saf..

[3] Zhang SN (2017). Talking about the formation mechanism and comprehensive prevention measures of hydrogen sulfide in coal mines. Energy Energy Conserv..

[4] Mao DQ, Hu CY (2008). The cause and prevention technology of hydrogen sulfide in coal mines. Sci. Technol. Inf..

[5] Liu MJ (2011). Discussion on the genetic types of hydrogen sulfide gas in coal mines. J. China Coal Soc..

[6] Deng QG (2019). Research advances of prevention and control of hydrogen sulfide in coal mines. Sci. World J..

[7] Deng QG, Liu MJ, Cui XF, Wen JJ (2017). Research on the genesis of hydrogen sulfide gas in coal mines in the southeastern margin of Junggar Basin. Earth Sci. Front..

[8] Tang WS (2016). Study on comprehensive treatment technology of hydrogen sulfide in Longmenxia North Mine. China Coal.

[9] Machel HG, Krouse HR, Sassen R (1995). Products and distinguishing criteria of bacterial and thermochemical sulfate reduction. Appl. Geochem..

[10] Cai CF (2009). Distinguishing Cambrian from Upper Ordovician source rocks: evidence from sulfur isotopes and biomarkers in the Tarim Basin. Org. Geochem..

[11] Zhu GY, Zhang SC, Liang YB, Dai JX (2006). The origin and prediction of high H_2_S content in natural gas. Chin. J. Geol..

[12] He Y, Fu XH, Lu L (2015). Analysis of factors affecting the adsorption of H_2_S by different coal ranks. Coal Mine Saf..

[13] He Y, Fu XH, Lu L (2016). Analysis of the difference in the adsorption of H_2_S, CH_4_ and N_2_ by coal. Chin. Sci. Technol. Pap..

[14] Liang B, Qu R, Sun WJ, Jia LF, Shi ZS (2016). Quantum chemical analysis of H_2_S gas adsorption characteristics on coal surface. J. Liaoning Tech. Univ..

[15] Xue JZ, Fu XH, Wu JH, Ding YM (2017). Adsorption characteristics of H_2_S gas in coal mine gas and its influence on governance. Geol. Prospecting.

[16] Xue JZ, Fu XH, Fan CJ, Song DY (2016). The adsorption difference and adsorption model of H_2_S gas by different coal rank coals. Coal Field Geol. Explor..

[17] Skoczylas N, Pajdak A, Kudasik M, Braga LTP (2020). CH_4_ and CO _2_ sorption and diffusion carried out in various temperatures on hard coal samples of various degrees of coalification. J. Nat. Gas Sci. Eng..

[18] Hou J, Wu J, Zhou K (2016). Distinct performance characters and inducing mechanisms of CO_2_ and N_2_ enhanced coalbed methane recovery. Int. J. Oil Gas Coal Technol..

[19] Gao DM, Hong L, Wang JR, Zheng D (2020). Molecular simulation of gas adsorption characteristics and diffusion in micropores of lignite. Fuel.

[20] Ma L, Li ZB, Deng J, Li B, Hu AP (2015). Study on the adsorption characteristics of N_2_/CO_2_/CH_4_ single component gas by coal under normal pressure. J. Saf. Environ..

[21] Wu SY, Deng CB, Wang XF (2019). Molecular simulation of flue gas and CH_4_ competitive adsorption in dry and wet coal. J. Nat. Gas Sci. Eng..

[22] Wu SY, Jin ZX, Deng CB (2019). Molecular simulation of coal-fired plant flue gas competitive adsorption and diffusion on coal. Fuel.

[23] Gao DM, Wang JR, Hong L, Zheng D (2019). The characteristics of gas adsorption in micropores and its influence on coal and gas outburst. Fresenius Environ. Bull..

[24] Zhang SJ, Li CW, Ding C, Zhang H (2010). Coal surface molecular fragment model and gas adsorption molecular mechanics simulation. Mining Res. Dev..

[25] Xiang JH, Zeng FG, Liang HZ, Li B, Song XX (2014). Molecular simulation of the adsorption of CH_4_/CO_4_/H_2_O in the molecular structure of coal. Sci. China Earth Sci..

[26] Gao DM, Hong L, Wang JR, Zheng D (2019). Adsorption simulation of methane on coals with different metamorphic grades. AIP Adv..

[27] Li YG (2019). Coal HRTEM image processing technology based on neural network. Coal Technol..

[28] Zhang KF (2020). Molecular simulation of the adsorption characteristics of methane in Zhaozhuang 3# coal. Chin. Sci. Technol. Pap..

[29] Li SG (2019). Molecular simulation of adsorption of gas in coal slit model under the action of liquid nitrogen. Fuel.

[30] Karasawa N, Goddard WA (1992). Force fields, structures, and properties of polyvinylidene fluoride crystal. Macromolecules.

[31] Li SG (2016). Molecular dynamics study of CO_2_ sorption and transport properties in coal. Fuel.

[32] Nosé S (1991). Constant temperature molecular dynamics methods. Prog. Theor. Phys. Suppl..

[33] Jin SQ (2011). H_2_S management in 15 # coal seam of Fenghuangshan coal mines. Procedia Eng..

[34] Liu ZX, Feng ZC (2012). Theoretical study on adsorption heat of methane in coal. J. China Coal Soc..

[35] Zhou F (2015). Effects of coal functional groups on adsorption microheat of coal bed methane. Energy Fuels.

